# Quality by design for transient RBD-Fc fusion protein production in Chinese hamster ovary cells

**DOI:** 10.1016/j.btre.2025.e00882

**Published:** 2025-02-09

**Authors:** Araya Jivapetthai, Wanatchaporn Arunmanee, Natapol Pornputtapong

**Affiliations:** a Department of Biochemistry and Microbiology, Faculty of Pharmaceutical Sciences, Chulalongkorn University, Bangkok 10330, Thailand; b Center of Excellence in Cancer Cell and Molecular Biology, Faculty of Pharmaceutical Sciences, Chulalongkorn University, Bangkok 10330, Thailand; c Center of Excellence in Systems Biology, Faculty of Medicine, Chulalongkorn University, Bangkok 10330, Thailand

**Keywords:** Box-Behnken, Fusion protein, Quality by design, RBD-Fc, Response surface methodology, SARS-CoV-2, Transient expression

## Abstract

Quality by design (QbD) is applied to the upstream process to maximize the RBD-Fc fusion protein production in CHO cells. The three factors (culture duration, temperature, and polyethyleneimine to plasmid DNA (PEI-Max/pDNA) ratio) were identified as critical process attributes based on risk analysis (FMEA) and further optimized by response surface to maximize the protein yields. Using a Box-Behnken design, the optimal conditions for RBD-Fc production were determined to be a culture duration of 5 days, a culture temperature of 34.4 °C, and a PEI-Max/pDNA ratio of 4.2:1 (w/w) with a predictive value of 48 mg/L (desirability of 92.8 %). The PEI-Max/pDNA ratio and its interaction with culture duration to express the highest yield (47.78 ± 2.30 mg/l). In addition, the purified CHO-produced RBD-Fc fusion protein was highly pure and strongly bound to its receptor, ACE2. Our finding demonstrated that the QBD tools can identify the critical parameters to facilitate scaling-up production.

## Highlights

• Quality by design can be applied to the upstream process of the fusion protein (RBD-Fc) to increase the production yield.
• Understanding the factors that affect product quality is essential for new findings to enhance process performance and maintain product quality.
• The process parameters are optimized by surface response methodology to find the optimum point to enhance production performance.
• A robust cell culture platform in the upstream process can ensure that critical quality attribute profiles are consistent from the early stages of laboratory-scale development to upscaling.

## Graphical abstract

**Figure:**
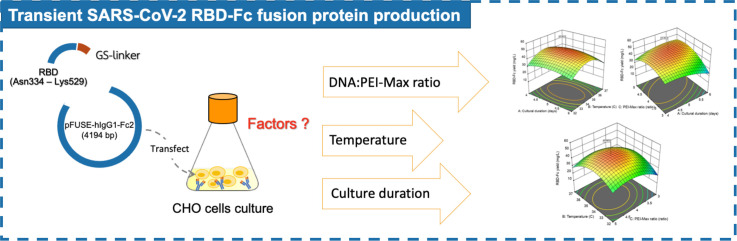

## 1 Introduction

Due to the emergence of COVID-19, numerous vaccine candidates have been developed based on the structure of the SARS-CoV-2 virus to elicit an immune response [[1], [2], [3], [4], [5], [6], [7]]. The intrusion of the virus is mediated through the binding of the receptor-binding domain (RBD) represented on the top of the spike protein as a key viral protein and its specific receptor, angiotensin-converting enzyme II (ACE2) [[8], [9], [10], [11]]. RBD is a potential target for vaccine design [8,12]. In addition, the RBD fused with the Fc domain of human IgG (RBD-Fc) holds promise as an effective SARS-CoV-2 vaccine, eliciting a strong antibody response with safe activity in immunized mice, rabbits, and nonhuman primates [[13], [14], [15], [16], [17]]. The RBD-Fc fusion protein induced a higher neutralizing antibody titer than the RBD protein alone, as evidenced by pseudovirus neutralization and live virus-based microneutralization assays [14] as well as RBD (delta)-Fc vaccine could trigger a durable immune effect by heterologous boosting immunity [15]. These results suggested that Fc fusion significantly enhanced humoral and cellular immune responses to recombinant RBD immunogens. Furthermore, Fc fusion proteins have gained considerable attention in drug development owing to their advantages, including efficient protein purification, prolonging the half-life of proteins, improving protein folding, enhancing binding to antigen-presenting cells, and stimulating immune cell interactions via Fc receptors, thus boosting immunological responses [16,17]. This evidence supports the idea that the SARS-CoV-2 RBD-Fc fusion could be a promising candidate for further development as a subunit vaccine against COVID-19.

The emergence of new variants in many countries requires further development. Hence, developing an effective and safe vaccine to prevent a new wave of pandemics is an urgent global health issue [[18], [19], [20]]. Vaccine production is challenging because of the nature of biopharmaceutical products, which are sensitive to the production processes, starting materials, and process parameters. To address this, the quality by design (QbD) approach is implemented by the US-FDA and the International Conference on Harmonization (ICH) in Q8(R2) to provide a better understanding of the production process with a continued improvement concept [[21], [22], [23], [24]]. Over the last decade, QbD has been adopted in vaccine development. In the early stages of biopharmaceutical development, designing quality into the production process can be difficult because of a lack of understanding of the nature of the product and proper production conditions. To address this, research should be conducted rapidly using a short-term approach to develop the proposed process design.

Accordingly, a large majority of biological products in commercial biotechnology are manufactured via mammalian cell platforms, including subunit vaccines, which have the potential to produce recombinant proteins with complex post-translational modifications [25,26]. Productive cell lines, such as CHO cells, have been modified to grow in suspension. This is advantageous for large-scale production and can be transiently expressed to produce large amounts of recombinant proteins [[25], [26], [27], [28], [29]]. These cells have been widely used in vaccine production, providing high quality, high productivity, and low impurities in the target product, which affect product efficacy and safety [[26], [27], [28], [29], [30]].

Recently, advances in transient expression systems and cell culture modifications have resulted in high yields in a short period. However, the complexity of the transient expression of recombinant proteins in CHO cells is a critical step to clarify, especially the culture conditions and transfection factors such as transfection reagents, which might affect the consistency of the target product, resulting in low production yield. Polyethyleneimine 40 kDa (PEI-Max) is a promising transfection reagent investigated for serum-free CHO cell suspensions owing to its cost-effective agent for further upscaling applications [30,31]. However, the ratio of PEI-Max to plasmid DNA would be determined for successful transfection.

Recombinant protein quality attributes are sensitive to culture parameters, with expression time being a key factor in achieving high expression efficiency at harvest time [[29], [30], [31]]. According to Rouiller et al., QbD was used to characterize the cell culture process of Fc fusion proteins. They reported that the culture duration had the highest impact on the process and product-related impurities and variants [32]. In addition, culture temperature as a process parameter affects protein expression and impurities [28,33]. The two factors have also been reported to affect the total protein content of veterinary vaccine production in baby hamster kidney cells [34]. Furthermore, Nagashima et al. found that culture duration, pH, and temperature are critical process parameters for monoclonal antibody production in CHO cell cultures [35]. Hence, the potential factors, such as the ratio of PEI-Max to plasmid DNA and culture parameters, including culture duration and temperature influence on recombinant protein quality attributes, were investigated.

In this study, we aimed to apply Quality by Design (QbD) methodologies in the upstream process of culturing Chinese Hamster Ovary (CHO) cells for the transient production of SARS-CoV-2 RBD-fused human IgG1-Fc proteins as a subunit vaccine candidate to improve its productivity. Optimizing a transient mammalian expression system for recombinant proteins of lab-scale output is required in the first pipeline of vaccine candidate development. Due to the culture conditions and transfection factors affecting recombinant protein quality attributes, the PEI-Max to plasmid DNA ratio, culture duration, and temperature were studied. RBD-Fc proteins were produced in suspension CHO cells (ExpiCHO expression system) by transient expression and were characterized in the resulting products using QbD tools. Cultural factors related to product quality attributes, including transfection factors and process parameters, were identified and selected using risk assessment tools. The potential factors were determined by optimizing the design of experiments (DoE) and Box–Behnken. RBD-Fc fusion proteins were produced under different conditions and secreted into the culture media. The protein was purified using affinity chromatography. Protein characterization and identification were performed using sodium dodecyl sulfate-polyacrylamide gel electrophoresis (SDS-PAGE) and western blotting. The binding activity of the ACE2 receptor was determined using ELISA. The protein concentration was measured, and the yield was calculated. It can improve its productivity in transient recombinant RBD-Fc fusion protein production in a suspension CHO cell system by fine-tuning multiple parameters such as culture duration, culture temperature, and PEI-Max/plasmid DNA ratio. Finally, optimal conditions for RBD-Fc protein production were determined.

## 2 Materials and methods

### 2.1 Materials

The ExpiCHO-S™ cell line was purchased from Thermo Fisher Scientific. The Hycell TransFx-C transfection medium was purchased from HyClone Laboratories. The SARS-CoV-2 *rbd* gene (Asn334 – Lys529) with a (G_4_S)_3_-linker was synthesized by Twist Biosciences. The expression plasmid encoding the RBD-Fc fusion protein was constructed using pFUSE-hIgG1-Fc2 (InvivoGen, USA). PEI-Max-linear polyethyleneimine hydrochloride (MW 40,000) was purchased from Polysciences, Inc. Recombinant Human ACE2 protein was purchased from Abcam. CHO-based RBD-Fc standard was purchased from Invitrogen. Goat anti-Human IgG (Fc specific)-Peroxidase antibody was purchased from Sigma. All chemical reagents used in this study were of analytical grade. The experimental design and statistical analysis software was Design Expert v.13.0.2, Stat-Ease, Inc.

### 2.2 Cell line and cell cultivation

ExpiCHO-S™ cells (Thermo Fisher Scientific, Lot 2,089,864) were adapted in Hycell TransFx-C transfection medium (Hyclone Laboratories, Logan, Utah, United States) and maintained in vented Erlenmeyer non-baffled shake flasks (Corning, cat. 431,143). The cells were cultured at 37 °C in a humidified incubator with 8 % CO_2_ at a shaking speed of 120 rpm (Eppendorf® New Brunswick™ S41i, Eppendorf AG, Germany). The cells were subcultured every 3–4 d. Cell viability and density were determined using trypan blue dry exclusion (0.4 %) (Gibco, cat. 15,250,061).

### 2.3 Plasmid DNA

To produce the RBD-Fc fusion protein, the RBD gene encoded pFUSE-hIgG1-Fc2 (Invitrogen, United States) expression plasmid was constructed by Laotee et al. [36]. Briefly, the SARS-CoV-2 *rbd* gene (Asn334 – Lys529) with a (G4S)3-linker was synthesized by Twist Bioscience, United States and inserted into the pFUSE-hIgG1-Fc2 plasmid. The inserted gene was located between the IL2 signal sequence and the human IgG1 Fc gene. The expression plasmid amplified in *E. coli* was extracted using a QIAGEN maxi kit (Qiagen, Germany). The DNA concentration was measured and assessed using a Nanodrop One microvolume UV–vis spectrophotometer (Thermo Fisher Scientific, United States).

### 2.4 To identify critical process parameters by risk assessment

The quality target profiles of protein-based vaccines (RBD-Fc) for COVID-19 are defined in Table 1, which lists the quality attributes required in the final product [37,38]. We focus on the upstream process of the SARS-CoV-2 RBD-Fc fusion protein production in suspension CHO cells, specifically investigating a product-related substance. Hence, increasing RBD-Fc protein yield with high purity is a goal attribute for the production process evaluation that plays a vital role in achieving the desired quality attributes listed in Table 2.

**Table 1:** Quality target product profiles of protein-based vaccine (RBD-Fc) for COVID-19.

Categories	Target product profiles	Quality characteristics
Vaccine platform description	Proteins-based subunit vaccine	Containing only the antigenic parts of the pathogen
Type of vaccine	Receptor binding domain (RBD) fused with human IgG1 Fc	As key viral protein and can bind to ACE2 and to elicit a protective immune response
Coronavirus target	SARS-CoV-2	Specific to SARS-CoV-2
Indication for use	Use for COVID-19 prevention	For active immunization of at-risk persons to prevent COVID-19
Target population	All ages	Critical to adults, and elderly
Route of administration	Injection	Liquid for injection
Safety and stability	Excellent stability with a safe profile	Safe and more stable Do not contain live components No risk of inducing the disease
Activity/Function	Effective immune response	Can produce an effective immune response

**Table 2:** Critical quality attributes for RBD-Fc protein production in CHO cells.

Category	Critical attributes	Design criteria
Product/Quantitation of antigenic content	Recombinant receptor binding domain fused with human IgG1 Fc (RBD-Fc) protein yield	To increase a higher yield of purified RBD-Fc protein (>5 mg/l)
Identity	Antigenic subunit in product	Correct size (dominant bands of RBD-Fc at 150 kDa in non-reducing and 50 kDa in reducing condition) Bound to anti human IgG1 Fc and anti-SARS-CoV-2 RBD antibody
Purity	High purity	Dominant bands of RBD-Fc at 150 kDa in non-reducing and 50 kDa in reducing condition by SDS-PAGE and Western blot without unspecific bands
Potency	Immunogenicity/Affinity activity with ACE2 receptor	Bound to human ACE2 receptors by ELISA

As cell culture directly impacts production efficiency and product quality, identifying the potential parameters that can affect the desired quality attribute for RBD-Fc protein production in CHO cells was initially performed using risk assessment-based tools [39]. The essential factors, including the material attributes and process parameters, were identified using an Ishikawa diagram. The diagram, including the branches of unit operations and their relevant factors, was drawn by brainstorming with the research team members subjected to low protein yield.

Then, all factors were scored and ranked into risk priority numbers (RPN) derived from prior knowledge and initial experimental data by failure mode and effect analysis (FMEA). Each factor was assessed in terms of three risks: severity of impact (S), probability of occurrence (O), and likelihood of detection (D) in Table 3. All terms were ranked on a five-level scale with a score of 1–5. A score of 5 indicates that the parameters have the highest impact on critical quality attributes. Each risk priority number (RPN) was calculated by multiplying the scores in Table 3, Table 4. The team members determined the RPN threshold, and the risk acceptance level was set to 50 according to the risk matrix in Table 4. High-risk factors were selected for the design of experiments (DoE) study to evaluate the impact of these potential factors on production yield.

**Table 3:** Failure mode effect analysis for critical process parameters determination.

Score	Definition
Severity of Impact (S)	
1	Not affect CQAs because we already have data
2	Not expected to affect CQAs based on experience and prior knowledge
3	Might affect CQAs based on experience and prior knowledge, and literature
4	Expected to affect CQAs based on experience and prior knowledge
5	Effects CQAs because we already have data
Probability of occurrence (O)	
1	Never happen in the foreseeable future (0 %)
2	Never but might happen (<1 %)
3	Rarely (below 5 %)
4	Sometimes (50 %)
5	Often (over 50 %)
Likelihood of detection (D)	
1	Change can be detected and dealt with immediately.
3	Change can be detected and dealt with within a day.
5	Change cannot be detected and dealt with.

**Table 4:** Table of a risk priority number (RPN) matrix.

Each risk priority number (RPN) was calculated by multiplying the scores for severity of impact (S), probability of occurrence (O), and the likelihood of detection (D). Risk colors: red: high impact; yellow: moderate; green: low impact. The RPN threshold was determined by the operator and the acceptance level of risk was set at 50 (in red).

### 2.5 Design of experiment

A design experiment (DoE) was used to design various experimental conditions to study the impact of the potential process parameters on RBD-Fc production. A Box-Behnken design was used to optimize the process variables. Potential factors were screened at three levels: low (coded as −1), medium (coded as 0), and high (coded as +1). The response function (Y) was partitioned into linear, quadratic, and interactive components. Thus, a mathematical model was established. The experimental data were used in non-linear regression analysis by fitting to a second-order polynomial equation Eq. (1) where b_0_ is the intercept; b_1_, b_2_, and b_3_ are linear coefficients; b_12_, b_13_, and b_23_ are interaction coefficients; and b_11_, b_22_, and b_33_ are squared coefficients.

(1) $$Y=b_{0}+b_{1}X_{1}+b_{2}X_{2}+b_{3}X_{3}+b_{12}X_{1}X_{2}+b_{13}X_{1}X_{3}+b_{23}X_{2}X_{3}+b_{11}X_{1}^{2}+b_{22}X_{2}^{2}+b_{33}X_{3}^{2}\ldots$$

### 2.6 Transfection and transient expression of recombinant RBD-Fc in CHO cells

The RBD-Fc fusion protein was transiently expressed in an ExpiCHO-S cell suspension. Briefly, recombinant plasmid DNA (pFUSE-hIgG1-Fc2 encoding the RBD gene) was transfected into ExpiCHO-S cells using the transfection reagent PEI-Max (Polysciences Inc. Cat. 24,765–1). The cultures were maintained under conditions different from the experimental design, as shown in Table 7. Before transfection, cell viability was >95 %, and the cells were seeded at 5.0 × 10^5^ cells/ml in 30 ml of culture medium and then maintained in 125 ml vented Erlenmeyer non-baffled shake flasks (Corning, cat. 431,143). The cells were allowed to grow overnight at a final density of 1.0 × 10^6^ cells/ml. For transfection (on day 0), PEI-Max solution and recombinant plasmid DNA were diluted separately with fresh media to a final concentration of 3–5 µg/ml and one ug/ml (PEI-Max/pDNA ratio (w/w), 3:1–5:1), respectively. Diluted PEI-Max was added to the diluted DNA solution and incubated at room temperature for 10 min. The DNA/PEI-Max complex was slowly added to the cell culture suspension. The culture was maintained at different temperatures, 32–37 °C which were adjusted on day 1 after transfection in a humidified incubator with the other constant parameters of 8 % CO_2_ and 120 rpm shaking speed (Eppendorf® New Brunswick™ S41i, Germany). The culture was harvested at different time intervals, 4–6 days.

### 2.7 Purification of CHO-expressed SARS-CoV-2 RBD-Fc fusion protein

On the day of protein harvesting, the cultures were harvested and clarified by centrifugation at 5,000x*g* at 4 °C for 30 min. The supernatant was filtered using a 0.22 µm syringe filter (Merck, Massachusetts, United States). The clarified supernatant was purified using affinity chromatography on a HiTrap™ FF MabSelect™ Prism A column (Cytiva, GE Healthcare Life Sciences, MA, United States) in Fast Protein Liquid Chromatography (FPLC) ÄTKA start (GE Healthcare, United States). Before purification, the column was equilibrated with 20 mM sodium phosphate buffer and 150 mM NaCl (pH 7.2). The filtered samples were injected into the column at a flow rate of 0.5 ml/min and then elution with 0.1 M sodium citrate buffer pH 3. Subsequently, the eluted fractions were pooled and neutralized with 1 M Tris–HCl buffer (pH 9). The purified protein was concentrated and exchanged in PBS buffer using an Amicon® Ultra-4 centrifugal filter unit-10 kDa cutoff (Merck, Darmstadt, Germany) and stored at −20 °C. The total protein content of the purified proteins was evaluated, and protein yield was determined.

### 2.8 Determination of protein yield

The concentration of purified proteins in each sample was measured using a bicinchoninic acid assay kit (Pierce™ BCA protein assay kit). Total proteins were calculated in mg of protein content in 30 ml of culture medium volume, as in Eq. (2). The purified SARS-CoV-2 RBD-Fc fusion protein yield was evaluated in mg/l, as in Eq. (3).

(2) Totalprotein(mg/30ml)=Proteinconcentration(mg/ml)xTotalvolume(ml)

(3) Yield(mg/l)=Totalprotein(mg/30ml)x1000(ml)

### 2.9 Protein characterization and identification

#### 2.9.1 Determination of RBD-Fc purity using SDS-PAGE and western blotting

After protein purification, the purified SARS-CoV-2 RBD-Fc fusion protein was detected using sodium dodecyl sulfate-polyacrylamide gel electrophoresis (SDS-PAGE) and western blotting under reducing and non-reducing conditions to identify the size and purity of the protein. RBD-Fc samples were loaded onto 10 % Tris-glycine sodium dodecyl sulfate-polyacrylamide gel electrophoresis and stained with Coomassie Brilliant Blue staining solution for protein band visualization. For western blot analysis, the separated proteins from the SDS-PAGE gel were transferred to a nitrocellulose membrane (Merck KGaA, Darmstadt, Germany) using a semi-dry transfer device, Trans-Blot® SD system, and PowerPac™ HC power supply system (Bio-Rad, Singapore). The nitrocellulose membrane was blocked by 5 % (w/v) skim milk in PBS buffer and detected with a 1:20,000 goat anti-Human IgG (Fc specific) - Peroxidase antibody (Sigma, St. Louis, MO, United States) diluted in 5 % (w/v) skim milk in 1xPBS buffer. The samples were probed using Immoblion® Forte Western HRP Substrate (Merck, Darmstadt, Germany). The bands were visualized using the chemiluminescent ImageQuant (LAS4000) program (GE Healthcare Technologist, United States). Additionally, an anti-RBD antibody was used to detect RBD-Fc bands using western blot analysis to confirm the identity of the RBD-Fc protein. Proteins separated by SDS-PAGE were transferred onto a nitrocellulose membrane. The membrane was blocked with 5 % (w/v) skim milk in 1xPBS buffer and detected with a 1:20,000 mouse anti-RBD antibody (R&D System, United States) diluted in 5 % (w/v) skim milk in 1xPBS buffer. The samples were probed using a 1:200,000 HRP-conjugated goat anti-mouse antibody and visualized using a chemiluminescent ImageQuant (LAS4000) machine.

#### 2.9.2 Binding assay with ACE2 using ELISA

The biological activity of RBD-Fc with its receptor, enzyme-linked immunosorbent assay (ELISA), was used to detect the binding between RBD-Fc and human ACE2. Briefly, a Nunc-Immuno MaxiSorp plate (Thermo Fisher Scientific, United States) was coated with 50 ng of human ACE2 (Abcam, United Kingdom) in 50 mM bicarbonate buffer (pH 9.6) at 4 °C overnight. The plate was blocked using a blocking buffer (PBS containing 1 % BSA) at 37 °C for 1 h. The plate was performed 3 times with PBST (PBS containing 0.05 % tween-20). Purified RBD-Fc fusion protein samples and a CHO-based RBD-Fc standard control (Invitrogen, United States) were prepared in two-fold serial dilutions, added to the wells, and incubated at 37 °C for 1 h. After washing, rabbit anti-human IgG (Fc specific) -peroxidase antibody (Sigma-Aldrich, United States) was added to the plates at a 1:1000 dilution in blocking buffer and incubated for 1.5 h at 37 °C. Subsequently, 3,3′,5,5′-tetramethylbenzidine (TMB) (Invitrogen, United States) was added, incubated at room temperature for 20 min, and stopped with 1 M H_2_SO_4_. A microplate reader (CALIOstar, BMG Labtech, Germany) collected data at 450 nm.

#### 2.9.3 Data and statistical analysis

Design of experiments (DoE) and calculation of predicted data were conducted using Design Expert software version 13.0.2 (Stat-Ease Inc., Minneapolis, United States). A Box-Behnken design (BBD) was used to design the experimental conditions for RBD-Fc protein production in CHO cells. An established model was created to evaluate the effects of each independent variable on the responses. The analysis of variance (ANOVA) and fitness of the polynomial model equation was expressed using the coefficient of determination *R^2^.* The statistical significance of the regression coefficients was considered significant at a significance level of 0.05 (*P* < 0.05). The optimized conditions and predicted values were verified in triplicate to confirm the model's validity.

## 3 Results and discussion

### 3.1 Determination of critical quality attributes (CQAs) and critical process parameters (CPPs) for RBD-Fc protein production in CHO cells

Developing an efficient production process to deliver consistent outcomes is required in the biopharmaceutical industry. The quality by design (QbD) approach provides a systematic guideline focusing on process development and attempts to predict a robust process. Experimentation can be performed using QbD tools based on risk management and design of experiment (DoE). However, the transient expression of recombinant proteins in suspension CHO cells has many critical steps to clarify, especially the related culture and transfection factors that might affect the consistency of SARS-CoV-2 RBD-Fc fusion protein production, resulting in low production yield.

**Fig. 1:**
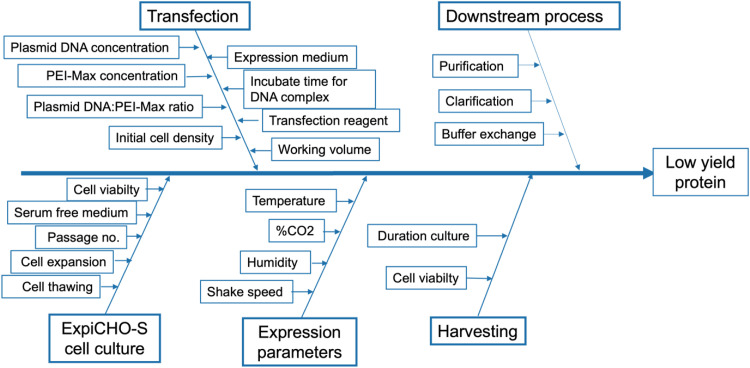
This is an Ishikawa diagram summarizing various potential factors that impact the yield of RBD-Fc fusion protein as a quality attribute in the suspension CHO cell production process. Input factors were categorized into five main groups: four in the upstream process and one in the downstream process.

**Fig. 2:**
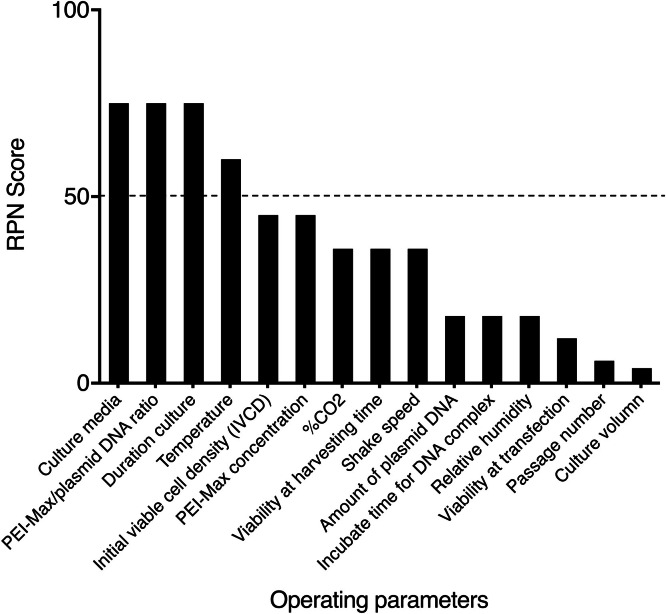
Pareto chart for the operating parameters in RBD-Fc protein production. RPN scores were determined using FMEA. The dash-line represents an RPN score of 50. Operating parameters with an RPN score higher than 50 were characterizing.

According to the QbD, the CQAs of the desired protein (RBD-Fc fusion) and CPPs were determined by a research team brainstorming using risk-based analysis following ICH Q9 guidance [39]. The various factors that influence RBD-Fc fusion protein production in suspension CHO cell culture are shown in the Ishikawa diagram in Fig. 1. The low yield of the RBD-Fc fusion protein is defined as a critical quality attribute in the suspension CHO cell production process. Input factors are categorized into five-unit operations: four in the upstream processes and one in the downstream processes. The upstream process is a part of the culture process, including CHO cell culture, protein expression, transfection, and harvesting, whereas the downstream process is the purification step. This study focused on the upstream process with four steps and 15 process parameters. The 15 parameters were given a score and are calculated in Table 5. The four parameters with an RPN greater than 50 are defined as potential critical variables in Fig. 2.

**Table 5:** Risk priority numbers for critical process parameters determination.

Materials/Process parameter	Unit	Pre-defined	S	O	D	RPN
Culture media	ml	30	5	3	5	75
Viability at transfection	%	>95	4	3	1	12
Viability at harvesting time	%	>70	3	4	3	36
Initial viable cell density (IVCD)	x 10^6^ cell/ml	1	5	3	3	45
Passage number	passage	1 – 20	3	2	1	6
Working volume	ml	30	4	1	1	4
Amount of plasmid DNA	ug/ml	1	3	2	3	18
PEI-Max concentration	ug/ml	<10	5	3	3	45
PEI-Max/Plasmid DNA ratio	ratio	1:3	5	3	5	75
Incubate time for DNA complex	min	10	3	2	3	18
Shake speed	rpm	120 ± 5	4	3	3	36
Temperature	°C	37	5	4	3	60
% CO2	%	8	4	3	3	36
Relative humidity	%	>80	3	2	3	18
Duration culture	Days	5	5	3	5	75

As the culture medium was identified as a critical material attribute for RBD-Fc fusion protein production in CHO cell culture, Hycell TransFx-C transfection medium was used as a constant variable throughout this study. This medium was chosen based on a report to achieve a high titer of recombinant protein (ßgal) expression in transient transfection when used with transfection reagent (PEI) in multiple CHO clones [40]. This can be used in a single medium to promote cell growth and transient transfection in CHO suspension culture, as this prompt medium would be helpful for the following purpose of transfer to large-scale production using stable expression. In the present study, we used this medium with PEI-Max (MW 40,000) to promote transfection. PEI-Max is a cationic polymer with less cytotoxicity [31,41,42] that can be applied in serum-free media and is compatible with this medium to enhance protein expression in CHO culture [30,31]. It increased the yield by >7-fold (4 to 28 mg/l) compared to the culture media for ExpiCHO cells in the preliminary study before the optimization process.

Accordingly, three potential parameters (culture duration, temperature, and PEI-Max/plasmid DNA ratios) were evaluated as critical process variables (CPPs) that can impact production yield based on risk analysis. First, the culture duration or expression time is one of the essential factors that allows the protein to be secreted into the culture media and achieve the highest expression efficiently at harvesting [32,34,35]. To define the setting range, we referred to the pilot condition for protein expression, which was set for 5 days at 37 °C using a 1:3 PEI-Max/plasmid DNA ratio (w/w). This study observed the culture duration for 4–6 days after transfection. Second, as a process parameter, culture temperature directly impacts protein expression and impurities related to cell culture [28,33,34]. The cells are maintained at a normal temperature of 37 °C to promote cell growth. However, some reports show that temperature reduction can increase the production yield in mammalian cultures [28]. Therefore, we investigated whether reducing the temperature from 37 °C to 32 °C would affect the protein yield. Lastly, the concentration of PEI to plasmid DNA (5:1 w/w) influenced transfection efficiency in CHO-3E7 cells for monoclonal antibody production. For this purpose, the difference between PEI-Max to plasmid DNA ratios between 3:1 and 5:1 was investigated to determine the optimal ratio for transfection to enhance the protein yield [43].

### 3.2 Optimization of the RBD-Fc production in CHO cells using response surface methodology

#### 3.2.1 Design of experiment of RBD-Fc production in CHO cells culture by a Box-Behnken

To determine the interaction effect of the three factors, the design of the experiment (DoE) was conducted using response surface methodology (RSM) in the Design-Expert program. The three input variables (culture duration, temperature, and PEI-Max/plasmid DNA ratio) were designed using a Box-Behnken design (BBD) at three levels, as shown in Table 6. The predefined range was set based on the preliminary results. The culture duration (A: 4–6 days), temperature (B: 32–37 °C), and PEI-Max/plasmid DNA ratio (C: 3:1–5:1 w/w) were assigned as independent variables, whereas the yield of the RBD-Fc fusion protein was defined as the response variable (Y: mg/l). A total of 17 experiments at different levels of the three-factor combinations in the actual and coded values are shown in Table 7. The cells were maintained under various culture conditions established using BBD design to determine their impact on RBD-Fc protein yield. Experimental results regarding the RBD-Fc protein yield (mg/l) were obtained. The observed yields were used as data to generate a predictive model to determine the optimum conditions for these factors to maximize the protein yield. The significance of the quadratic term was preferable to enable the model to navigate the optimal range that agrees with the confirmative results within the acceptance range.

**Table 6:** Independent variables selected for Box-Behnken design optimization.

Independent variables	Level			Dependent variables (Y)	Goal
	−1	0	+1		
A: Duration culture (days)	4	5	6	Y1: Yield (mg/l)	Maximized
B: Temperature ( °C)	32	34.5	37		
C: PEI-Max/pDNA ratio (w/w)	3:1	4:1	5:1		

The three levels (−1, 0, 1) of factor A (Duration) represented 4, 5, and 6 days, respectively. The three levels (−1, 0, 1) of factor B (Temperature) represented 32, 34.5, and 37 °C, respectively. The three levels (−1, 0, 1) of factor C (PEI-Max/pDNA ratio) represented 3:1, 4:1, and 5:1 w/w, respectively.

**Table 7:** Box-Behnken design and response values for RBD-Fc protein yield.

Run	Factor (Coded)			Actual variables			Yield (mg/l)		
	A	B	C	Duration (days)	Temp ( °C)	PEI-Max/ pDNA ratio (w/w)	Observed value (Yobs)	Predicted value (Ypred)	Residual
1	−1	0	−1	4	34.5	3	37.52	37.21	0.3113
2	0	1	1	5	37	5	28.65	27.70	0.9463
3	0	0	0	5	34.5	4	48.46	48.18	0.2840
4	1	−1	0	6	32	4	32.72	30.51	2.2100
5	1	0	−1	6	34.5	3	22.73	24.00	−1.2700
6	0	0	0	5	34.5	4	45.59	48.18	−2.5900
7	0	−1	−1	5	32	3	19.81	20.76	−0.9462
8	0	−1	1	5	32	5	30.52	32.42	−1.9000
9	1	1	0	6	37	4	27.98	28.61	−0.6350
10	1	0	1	6	34.5	5	42.01	42.32	−0.3112
11	−1	1	0	4	37	4	30.13	32.34	−2.2100
12	−1	0	1	4	34.5	5	35.32	34.05	1.2700
13	0	0	0	5	34.5	4	48.27	48.18	0.0940
14	−1	−1	0	4	32	4	32.36	31.72	0.6350
15	0	0	0	5	34.5	4	50.82	48.18	2.6400
16	0	1	−1	5	37	3	26.10	24.20	1.9000
17	0	0	0	5	34.5	4	47.74	48.18	−0.4360

Observed and predicted values for the quadratic model. A: Cultural duration (days); B: Temperature ( °C); C: PEI-Max to plasmid DNA ratio (w/w). Yield: a total of purified RBD-Fc protein content in 1 L of culture medium volume.

#### 3.2.2 Development of regression equation for prediction of RBD-Fc protein yield

As suggested by Design-Expert software, a quadratic model was the best-fit model to correlate the independent variables with the dependent variables. Form model summary statistics were used to check the adequacy of the models generated from the obtained data. The results are presented in Table 8. The model summary output showed that the R^2^, adjusted R^2^, and predicted R^2^ values for the quadratic model were nearest to 1 compared with the other models. The adjusted R^2^ value was 0.9450, indicating a high correlation between the observed (Y_obs_) and predicted R^2^ values (Y_pred_) for RBD-Fc protein yield, as shown in Table 7. Moreover, the adjusted R^2^ (0.9450) and predicted R^2^ (0.7460) had a difference of <0.2, which was required for the fit model and suitable for predicting the response. Additionally, a signal-to-noise ratio (15.5554) more significant than four is desirable for adequate precision measurements to confirm model fitting.

**Table 8:** Model summary statistics for production yields of RBD-Fc protein.

Source	Sequential p-value	Lack of Fit p-value	Adjusted R²	Predicted R²	Remarks
Linear	0.7592	0.0012	−0.1281	−0.4203	
2FI	0.7905	0.0007	−0.3273	−1.1908	
Quadratic	< 0.0001	0.2307	0.9450	0.7460	Suggested
Cubic	0.2307		0.9636		Aliased
*Fit statistics*					
Quadratic			R^2^	0.9759	Selected
Std.Dev.	2.30		Adjusted R^2^	0.9450	
Mean	35.69		Predicted R^2^	0.7460	
C.V. %	6.44		Adeq Precision	15.5554	

The predicted R^2^ of 0.7460 is in reasonable agreement with the adjusted R^2^ of 0.9450; the difference is <0.2. Adeq Precision measures the signal-to-noise ratio. A ratio greater than 4 is desirable. The ratio of 15.555 indicates an adequate signal. This model can be used to navigate the design space. C.V: coefficient variation. Std.Dev.: Standard deviation.

**Fig. 3:**
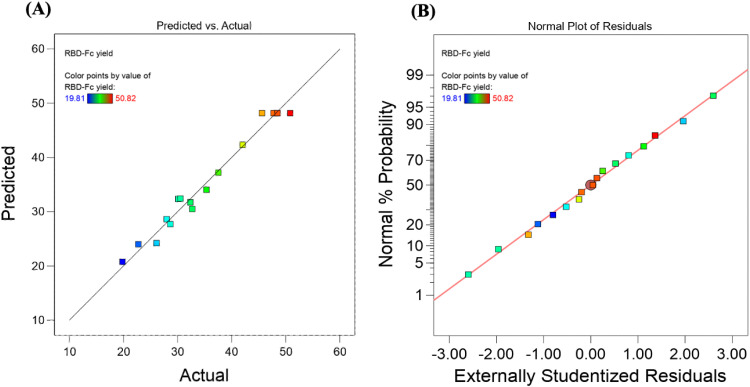
Model fitting illustrates the correlation of (A) Graphic representation of the observed values as a function of predicted values and (B) Normal probability plots of residuals for the yield of RBD-Fc protein.

According to the correlation graph shown in Fig. 3A, the observed and predicted values are correlated, and all residual values are within the acceptable range (<2σ), demonstrating a good correlation between the observed and predicted values. In addition, the residuals established a normal distribution that closely followed the fitted line with no reasonable outliers, as shown in Fig. 3B. Consequently, the quadratic model term can navigate through the output. The equation from the fitted quadratic model representing the purified RBD-Fc protein yield (mg/l) in terms of coded variables is as follows: Eq. (4).

(4) $$\text{Purified}\;\text{RBD}-\text{Fc}\;\text{protein}\;\text{yield}\left(\text{mg}/l\right)=48.18-1.24A-0.3187B+3.79C-0.6275\text{AB}+5.37\text{AC}--2.04\text{BC}-4.63A^{2}-12.75B^{2}-9.15C^{2.}\ldots$$

Where A, B, and C are the coded values of the independent variables, A is the culture duration (d), B is the temperature ( °C), and C is the PEI-Max to plasmid DNA ratio (w/w).

#### 3.2.3 Statistical analysis for selected models and analysis of variance

A quadratic model emerged as the best-fit model for BBD-based experimental design. Therefore, this regression model was further tested for its significance and adequacy using an ANOVA. A summary of the variance (ANOVA) for the quadratic polynomial model (Eq. (4)) is presented in Table 9. The ANOVA of the quadratic regression model demonstrated that the model was significant. At the same time, the lack of fit was not significant due to the variation and the inadequacy of the model. Therefore, there is no evidence that the model adequately explains the variation in response.

**Table 9:** Result of ANOVA for production yield of RBD-Fc protein.

Source	Sum of squares	df	Mean square	F-value	p-value	Remark
Model	1498.57	9	166.51	31.52	< 0.0001	Significant
A	12.23	1	12.23	2.31	0.1720	Not significant
B	0.8128	1	0.8128	0.1539	0.7065	Not significant
C	115.06	1	115.06	21.78	0.0023	Significant
AB	1.58	1	1.58	0.2982	0.6020	Not significant
AC	115.35	1	115.35	21.84	0.0023	Significant
BC	16.65	1	16.65	3.15	0.1191	Not significant
A²	90.13	1	90.13	17.06	0.0044	Significant
B²	684.66	1	684.66	129.62	< 0.0001	Significant
C²	352.84	1	352.84	66.80	< 0.0001	Significant
Residual	36.98	7	5.28			
Lack of Fit	23.02	3	7.67	2.20	0.2307	Not significant
Pure Error	13.96	4	3.49			
Cor Total	1535.55	16				

A: cultural duration (days); B: cultural temperature ( °C); C: PEI-Max to plasmid DNA ratio (w/w). df: degree of freedom; p-value<0.05 was significant.

### 3.3 Effect of culture duration, temperature, and PEI-Max/pDNA ratio on RBD-Fc protein yield

To determine the effect of the process parameters on the productivity of the RBD-Fc protein, the impact of the three-factor interaction on the RBD-Fc protein yield was investigated using response surface methodology. The significance of each coefficient was determined by p-values <0.05, indicating significant model terms. Based on the p-values for each coefficient in Eq. (4), the quadratic term coefficients of A^2^, B^2^, and C^2^, the interaction term coefficient of AC, and the linear term coefficients of C showed significant differences. In contrast, the other term coefficients were not significant.

**Fig. 4:**
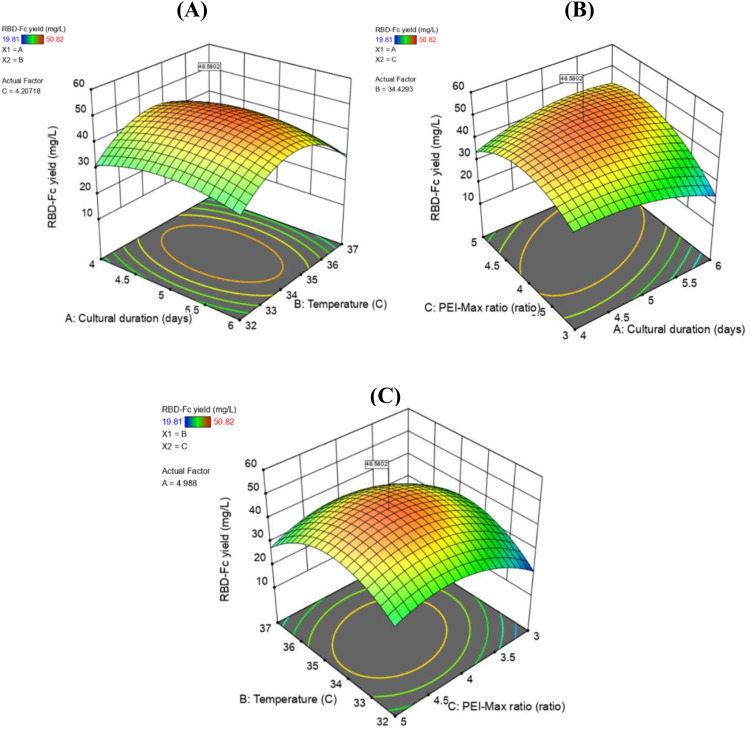
Response surface showing the effects of duration culture (A), Temperature (B), and PEI-Max/pDNA ratio (C) on the yield of RBD-Fc protein. (A) Response surface of yield as effect of cultural duration and temperature at a constant of 4.21:1 PEI-Max/pDNA ratio (w/w); (B) Response surface of yield as effect of cultural duration and PEI-Max/pDNA ratio at a constant of 34.4 °C of cultural temperature and (C) Response surface of yield as effect of temperature and PEI-Max/pDNA ratio at a constant of 4.99 days of cultural duration.

A 3D plot was generated for the models as a function of the two variables to visualize the relationship between the independent variables and responses. The 3D response surface is a graphical representation of the regression in Eq. (4). It exhibited a method for visualizing the relationship between the response and experimental levels of each variable and the type of interactions between the two test variables. The effects of culture duration, temperature, and PEI-Max/pDNA ratio on the RBD-Fc protein yield are shown in Fig. 4A–C. On the response surface, the yield was obtained along with the ratio of two independent variables on the x- and y-axes, and the response was shown on the z-axis, where the remaining factors were held constant at the optimum point. According to the 3D plot, the interaction between culture duration (A) and temperature (B), where the PEI-Max/pDNA ratio (C) was fixed at 4.21:1 ratio (w/w) (Fig. 4A). The interaction between culture duration (A) and PEI-Max/pDNA ratio (C), where the temperature (B) was fixed at 34.4 ( °C) (Fig. 4A). Interaction between temperature (B) and PEI-Max/pDNA ratio (C), where the culture duration was fixed at 4.9 (days) (Fig. 4C). The results showed that the yield first increased rapidly and then decreased with an increase in all three variables. The optimum ranges of the three parameters were in the center of their ranges. According to the regression coefficient significance of the quadratic polynomial model (Table 9) and the gradient of the slope in the 3D plot (Fig. 4A–C), the PEI-Max/pDNA ratio was the most significant factor affecting protein yield when the other factors, temperature, and culture duration were changed at each level.

However, production efficiency is not the effect of a single factor; the interaction of various factors in production affects the production process and achieves a high yield. The three potential factors showed an interaction effect on the yield and quality of the target protein. Among the three parameters tested, the ratio of PEI-Max to plasmid DNA was the most prominent factor affecting the efficiency of expression of the RBD-Fc fusion protein in CHO cells and its interaction with culture duration. Many studies have supported the influence of the variations and interactions of these potential parameters on recombinant protein expression in mammalian cells on the process and product-related impurities and variants. The PEI-Max/pDNA ratio works together with the expression time, initial cell density, and viability of the transient recombinant prothrombin system [42]. The interaction between duration and temperature affects the total protein content in baby hamster kidney cells for veterinary vaccine production [34]. Furthermore, culture duration, pH, and temperature combination have been reported as a critical process parameter for mAb production in CHO cell cultures [35].

### 3.4 Optimum condition of the CPP variables for RBD-Fc protein production in CHO cells

**Fig. 5:**
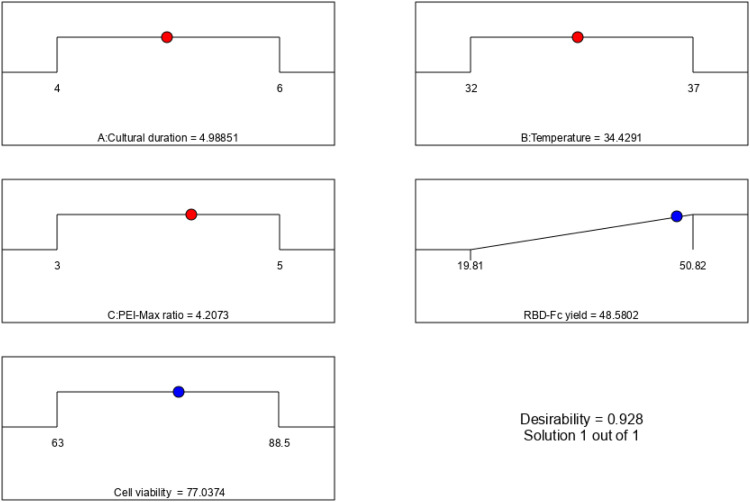
Desirability ramp for optimization of the RBD-Fc protein production in CHO cells. Three variables, including culture duration (4–6 days), temperature (32–37 °C), and PEI-Max/pDNA ratio (3:1–5:1 w/w), were set at the range value, whilst the RBD-Fc fusion protein yield was set at the maximum value. The prediction profile showed the desirability was close to 1 (desirability of 0.928) to provide the highest protein yield (48.58 mg/l).

RBD-Fc protein production in CHO cells was optimized using numerical optimization designed by Design-Expert software. This analysis considered the interaction between factors to determine the optimal conditions for a response using desirability functions. The variables and response were set as option goals (i.e., maximum, minimum, target, in range, or equal to), where all goals were then combined into one desirability function. To determine the optimum conditions that would meet all the goals, three variables, including culture duration (4–6 days), temperature (32–37 °C), and PEI-Max/pDNA ratio (3:1–5:1 w/w), were set at the range value. In contrast, the RBD-Fc fusion protein yield was set at the maximum value. For the importance of goals, all variables were set as equally important in score 3 (score 1–5), whereas the response variables were set at a score of 5 to maximize protein yield. By applying the desirability function approach, desirability ramps of the optimal point were developed from the optimum point via numerical optimization. According to Fig. 5, the prediction profile showed that the desirability was close to 1 (desirability of 0.928), and the highest protein yield was 48.58 mg/l. The optimum conditions were obtained based on the optimization study, as listed in Table 10. There were 4.99 d of culture time, 34.43 °C culture temperature, and a 4.21:1 ratio w/w of PEI-Max to plasmid DNA.

**Table 10:** Experimental confirmation of predicted value at optimal condition of RBD-Fc protein production in CHO cells.

Optimum level	Predicted mean (Y_pred_)	Experiment value (*n* = 3)			Observed mean (Y_obs_)	Std Dev	95 % PI low	95 % PI High
		N1	N2	N3				
*A* = 4.99 *B* = 34.43 C = 4.21:1	48.58	47.90	46.84	48.57	47.78	2.30	44.62	52.54

A: culture duration (days); B: culture temperature ( °C); C: PEI-Max/pDNA ratio (w/w).

The optimal points of these potential factors were in the center of their range using the predicted model. The results showed that reducing culture temperature from 37 to 34.5 °C under the optimum ratio (4.2:1 w/w) of PEI-Max/plasmid DNA influences protein expression to enhance protein yields in CHO culture for only 5 days after transfection. However, we suppose that the low culture temperature (32 °C) and high temperature (>37 °C) are not suitable for maintaining the culture; they can suppress the growth of cells and lead to increased aggregate protein [28,44]. It would be suggested that the temperature be reduced after the transfection day to allow cell growth before accumulating the protein yield.

Within the optimal range (Table 10), the ExpiCHO cell culture expressed an RBD-Fc protein yield of nearly 50 mg/l of culture volume, as high as 10-fold compared to previous results before optimization (approximately 5 mg/l). Compared to spike RBD transiently produced in the ExpiCHO system using a maximum titer condition, it has been reported to obtain a yield of 40 mg/l [45]. This finding indicates that utilizing mammalian cell lines based on the CHO system, along with an optimized process through quality-by-design methodology, improves the production of RBD-Fc protein and achieves high yields. Moreover, CHO cell-based production provides human-like post-translational modifications, unlike other systems such as plant-based production (*Nicotiana benthamiana*), which results in a lower RBD-Fc protein yield of approximately 22 mg/kg [46]. A large-scale yeast (*Pichia pastoris*) expression system produces around 21 mg/l of RBD, but there are notable differences in glycosylation patterns [47]. With the advancement of CHO cell line development and process optimization, most recombinant proteins expressed in CHO cells have achieved protein yields as high as milligrams to grams in a transient system [33]. However, the yield of RBD-Fc expressed from CHO culture was not as high as g/l, which could be a limitation to the synthesis and secretion of many complex recombinants. Low productivity, growth restrictions, expression instability, and high production costs are concerns. In addition, the intracellular protein processing complex can be involved in protein expression, including cellular secretion, protein aggregation or degradation, and protein folding, which could result in low protein expression or limited expression of recombinant proteins [27].

### 3.5 Experimental confirmation of the predictive models

The optimum conditions for the transient production of the RBD-Fc protein in CHO cells were intended to obtain the maximum protein yield and higher purity. To validate the model, experimental tests were conducted to confirm the prediction in triplicate under modified optimal conditions for practical operation. The condition was set for 5 days of expression time, 34.5 °C of cultural temperature, and a 4.2:1 ratio w/w of PEI-max to plasmid DNA. The purified RBD-Fc protein yield agreed with the predicted optimum value of 47.78 ± 2.30 mg/l, approximately equal to the predicted yield (48.58 mg/l), as shown in Table 10. In addition, the verification value for purified RBD-Fc protein was obtained within 95 % of predictive values (PI) at 44.62 mg/l and 52.54 mg/l for low PI and high PI, respectively, which clearly showed that the model fitted the observed data very well. Hence, optimized RBD-Fc production was efficiently achieved within the optimum range of process parameters.

### 3.6 Protein purification and characterization

**Fig. 6:**
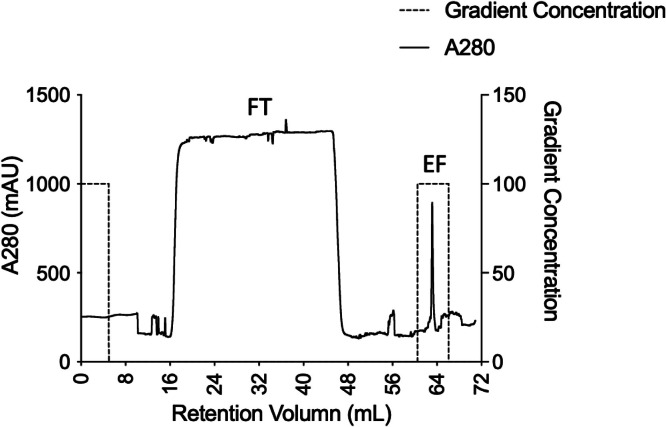
Elution profile for RBD-Fc protein purification using Fast Protein Liquid Chromatography. The purification was carried out by a HiTrap™ FF MabSelect™ PrismA column where RBD-Fc fusion protein is specifically bound to the protein A column. EF: Eluted fraction, FT: Flow-Through of unbound proteins.

As the IL-2 signal sequence presented in the expression vector, RBD-Fc proteins were secreted into the culture medium. On the day of harvesting, the supernatant was collected via centrifugation and filtered to remove the cell pellet. For purification, the filtered samples were loaded onto a HiTrap™ FF MabSelect™ Prism A column (Cytiva, GE Healthcare Life Sciences, MA, United States), a protein A affinity chromatography, in Fast Protein Liquid Chromatography (FPLC) ÄTKA start (GE Healthcare, United States). As the RBD fused with human IgG1-Fc was strongly retained in the column, impurities without IgG1-Fc were not bound to the column. The eluted fraction (EF) was the RBD-Fc protein that was eluted, as shown by the peak of EF in the chromatogram in Fig. 6. The flow-through fraction (FT) contained unbound proteins as impurities obtained during purification of the RBD-Fc protein. From the results, production under optimal conditions provides high-purity protein due to the presence of the human IgG Fc region linked to the RBD in the fusion protein, which offers favorable characteristics for improving protein solubility and stability to facilitate an effective purification step [48].

**Fig. 7:**
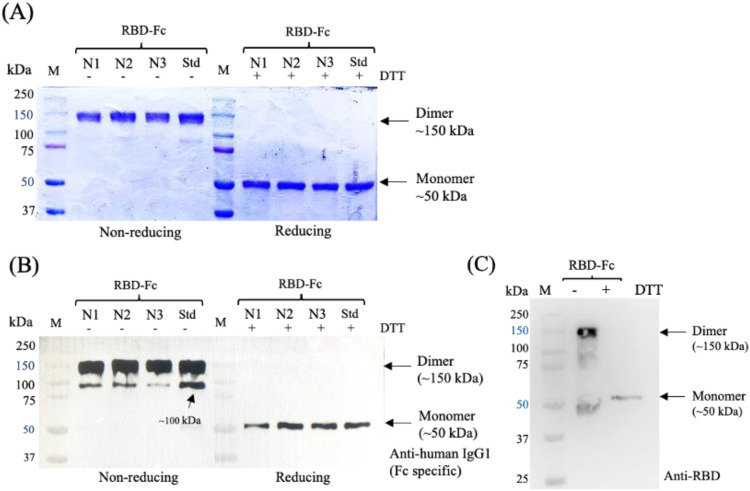
SDS-PAGE and Western blot analysis of RBD-Fc proteins expressed in ExpiCHO cell culture. (A) A Coomassie-stained SDS-PAGE of purified RBD-Fc samples was visualized on 10 % SDS-PAGE gel with non-reducing (-) and reducing (+). (B-C) Western blot analysis of purified RBD-Fc proteins was detected using the anti-human IgG1 Fc (B) and anti-SARS-CoV-2 RBD (C). Lane M: protein marker; Lane N1-N3: the protein samples were taken on the elution fraction after purification by affinity chromatography that were produced from the triplicate of confirmed experiments at optimal conditions; Lane Std: RBD-Fc produced in-house was used as a reference protein standard; DTT: Dithiothreitol (reducing agent).

Because purity is a critical quality attribute that affects protein yield, it must be assessed for any protein sample. After purification, SDS-PAGE and western blotting were performed to confirm the size and purity of the purified proteins. To evaluate the characteristics of the CHO-produced RBD-Fc, purified RBD-Fc was analyzed by SDS-PAGE. The visualized gel showed bands at approximately 50 and 150 kDa in reducing and non-reducing conditions, respectively (Fig. 7A). According to the theoretical size, the bands of the RBD-Fc protein are at 48.8 kDa and 120 kDa that represent monomeric and dimeric forms of RBD-Fc. As expected, the dimeric form of the RBD-Fc protein expressed in CHO cells was assumed to have a molecular weight of 120 kDa. However, we suggest that the glycans on RBD-Fc might affect its electrophoretic mobility on SDS-PAGE gels, compared to those of molecular weight markers, and cause a shift in the molecular weight readout from the gel. To confirm the presence of the protein, purified RBD-Fc samples were visualized by western blotting. All bands visualized by SDS-PAGE were detected using an anti-human IgG1-Fc antibody (Fig. 7B), and anti-RBD antibodies (Fig. 7C) under non-reducing and reducing conditions. Hence, these bands indicate that the RBD-Fc in this study comprised RBD and Fc domains that can specifically bind to its antibodies. Additionally, the observed protein bands, approximately 100 kDa in size, were also recognized by a detection antibody, suggesting that this species could be a variant of RBD-Fc presented in the samples.

### 3.7 Binding activity of CHO-produced RBD-Fc protein

**Fig. 8:**
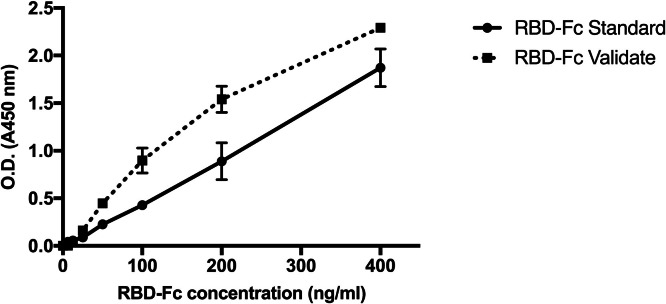
Binding activity of purified RBD-Fc protein to ACE2 receptor determined by ELISA. The dashed line represents the RBD-Fc protein sample, and the solid line represents the RBD-Fc protein standard.

According to, the biological activity of the RBD-Fc protein binds to its specific receptor, and the binding affinity of the RBD-Fc protein to its receptor, human ACE2, was determined by ELISA. Because of its specific binding to ACE2, the purified RBD-Fc (samples) and RBD-Fc commercial standard (InvivoGen) could bind to human ACE2 in a dose-dependent manner, as shown in Fig. 8. Various concentrations of purified CHO-produced RBD-Fc proteins were incubated with human ACE2 (Abcam, Cambridge, United Kingdom). Then, anti-human IgG (Fc-specific)-peroxidase antibody (Sigma-Aldrich, United States) followed by tetramethylbenzidine (TMB) (Invitrogen, United States) were added to the wells to detect the purified RBD-Fc protein. The results showed that the purified CHO-produced SARS-CoV-2 RBD-Fc fusion protein and the commercial RBD-Fc protein bound with substantial affinity to ACE2 proteins in a dose-dependent manner. These data indicated that the SARS-CoV-2 RBD-Fc fusion protein produced from CHO cells folded correctly and bound with commercial ACE2 proteins to maintain its antigenicity.

Recombinant protein production in mammalian cell culture processes involves many influencing factors that can be further characterized. To ensure that the practical operation to control strategies for RBD-Fc protein production does not affect the consistency of protein yield and quality, the condition should be performed under the defined optimal ranges of CPPs based on a culture duration of 5 days (120 ± 2 h) after transfection, 34.5 ± 0.5 °C at the culture temperature setting, and 4.2 ± 0.2:1 ratio (w/w) of PEI-Max to plasmid DNA for efficient transfection. Although CPPs can be accurately measured and maintained during manufacturing, other parameters should be controlled within their optimal ranges, which could impact product performance.

Eventually, a robust cell culture platform in the upstream process can ensure that critical quality attribute (CQAs) profiles are consistent from the early stages of laboratory-scale development to large-scale production. Although a basic understanding of how variations in common raw material attributes and cell culture process parameters affect the yield and quality of the desired protein is increasing, this knowledge is still far from complete enough to encompass the variation observed in cell culture, which may affect the expression of proteins, including the yield and quality of recombinant proteins. Nevertheless, understanding the factors that affect product quality is essential for new findings to enhance process performance and maintain product quality.

## 4 Conclusion

This study describes the successful application of QbD principles to process optimization for transient SARS-CoV-2 RBD-Fc protein production, which maximized production yield in CHO cell culture. The expression of SARS-CoV-2 RBD-Fc achieved the highest yield (48 mg/l) within five days post-transfection under an optimal range of factors in CHO cell suspension culture. Attributes such as culture duration, temperature, and polyethyleneimine (PEI-Max) to plasmid DNA ratio were identified as critical process attributes. Among the three parameters tested, the ratio of PEI-Max to plasmid DNA was the most prominent factor affecting the efficiency of RBD-Fc protein expression in CHO cells and its interaction with the culture duration and temperature. Using the Box-Behnken design, the optimum conditions for RBD-Fc fusion protein production were 5 days of culture time, 34.4 °C culture temperature, and a 4.2:1 ratio w/w of PEI-Max to plasmid DNA. Our results demonstrated that the effective RBD-Fc fusion protein can transiently produce the highest yield under established optimum conditions for CHO cell culture implemented by QbD methodologies and can be further developed in pilot settings for application in the initial stage of scale-up development.

## Funding

This work was supported by the Government Pharmaceutical Organization (GPO) grant (02/2564) Thailand and the Thailand Science Research and Innovation Fund Chulalongkorn University. The authors are grateful for the 100th anniversary of the Chulalongkorn University Fund.

## CRediT authorship contribution statement

**Araya Jivapetthai:** Writing – review & editing, Writing – original draft, Methodology, Investigation, Data curation. **Wanatchaporn Arunmanee:** Writing – review & editing, Writing – original draft, Supervision, Resources, Investigation, Funding acquisition, Conceptualization. **Natapol Pornputtapong:** Writing – review & editing, Writing – original draft, Supervision, Resources, Investigation, Funding acquisition, Conceptualization.

## Declaration of competing interest

The authors declare that they have no known competing financial interests or personal relationships that could have appeared to influence the work reported in this paper.

## Data Availability

Data will be made available on request.

## Appendix Supplementary materials

Image, application 1
