# Supplementary material for: Quality by design for transient RBD-Fc fusion protein production in Chinese hamster ovary cells
Source: Biotechnol Rep (Amst). 2025 Feb 9;45:e00882. doi: 10.1016/j.btre.2025.e00882 (PMC11872631; doi:10.1016/j.btre.2025.e00882)
Supplement: Supplementary file 1 [file mmc1.docx]

**Supplementary Materials**

**Binding activity with ACE2 receptor determined by ELISA**

**OD 450 nm. of RBD-Fc standard and validate sample in 2-fold dilutions**

| **A450** | **[RBD-Fc] Standard (ng/ml)** | | | | | | | **Blank** |
| --- | --- | --- | --- | --- | --- | --- | --- | --- |
|  | **400.0** | **200.0** | **100.0** | **50.0** | **25.0** | **12.5** | **6.25** |  |
| OD1 | 2.266 | 1.246 | 0.655 | 0.430 | 0.261 | 0.231 | 0.246 | 0.155 |
| OD2 | 2.020 | 1.098 | 0.604 | 0.431 | 0.268 | 0.238 | 0.203 | 0.183 |
| OD3 | 1.872 | 0.861 | 0.566 | 0.360 | 0.275 | 0.239 | 0.221 | 0.201 |
| **Average** | **2.053** | **1.068** | **0.608** | **0.407** | **0.268** | **0.236** | **0.223** | **0.180** |
| **S.D.** | **0.199** | **0.194** | **0.045** | **0.041** | **0.007** | **0.004** | **0.022** | **0.023** |
| **OD -blank** | **1.873** | **0.889** | **0.429** | **0.227** | **0.088** | **0.056** | **0.044** | **0.000** |
| **A450** | **[RBD-Fc] Validate (ng/ml)** | | | | | | | **Blank** |
|  | **400.0** | **200.0** | **100.0** | **50.0** | **25.0** | **12.5** | **6.25** |  |
| OD1 | 2.512 | 1.882 | 1.251 | 0.665 | 0.373 | 0.243 | 0.207 | 0.229 |
| OD2 | 2.530 | 1.741 | 1.051 | 0.674 | 0.358 | 0.236 | 0.196 | 0.196 |
| OD3 | 2.445 | 1.608 | 1.000 | 0.612 | 0.361 | 0.250 | 0.214 | 0.184 |
| **Average** | **2.496** | **1.744** | **1.101** | **0.650** | **0.364** | **0.243** | **0.206** | **0.203** |
| **S.D.** | **0.045** | **0.137** | **0.133** | **0.034** | **0.008** | **0.007** | **0.009** | **0.023** |
| **OD -blank** | **2.293** | **1.541** | **0.898** | **0.447** | **0.161** | **0.040** | **0.003** | **0.000** |

A450: Absorbance measurement at 450 nm.; Blank: Phosphate buffer saline (PBS); OD: Optimal density; S.D: Standard deviation
